# Protocol for mapping neural circuit connectivity with START: Single transcriptome assisted rabies tracing

**DOI:** 10.1016/j.xpro.2026.104634

**Published:** 2026-06-19

**Authors:** Maribel Patiño, Sai Krishna Bhamidipati, Christian Cazares, Edward M. Callaway

**Affiliations:** 1 Systems Neurobiology Laboratory, The Salk Institute for Biological Studies, La Jolla, CA, USA; 2 Department of Psychiatry, University of California, San Diego, La Jolla, CA, USA; 3 Department of Neurobiology, School of Biological Sciences, University of California, San Diego, La Jolla, CA, USA; 4 Department of Cognitive Science, University of California, San Diego, La Jolla, CA, USA

**Keywords:** RNAseq, model Organisms, Molecular Biology, Neuroscience

## Abstract

Characterizing neural connectivity at transcriptomic cell-type resolution is essential for understanding neural mechanisms of circuit function. Here, we present single transcriptome assisted rabies tracing (START), a protocol combining monosynaptic rabies tracing with single-nucleus RNA sequencing to identify transcriptomic cell types providing inputs to defined neuronal populations in the mouse cortex. We describe steps for Cre-dependent helper virus injection, EnvA-pseudotyped rabies infection, tissue microdissection, and fluorescence-activated nuclei sorting. We then detail procedures for library preparation and computational annotation of nuclei.

For complete details on the use and execution of this protocol, please refer to Patiño et al.^1^

## Highlights

• Protocol to map neural connectivity at transcriptomic resolution using START
• Steps for monosynaptic rabies tracing in genetically defined starter cells
• Instructions for nuclei isolation and FACS sorting of rabies-labeled neurons
• Pipeline for transcriptomic cell-type annotation of presynaptic input neurons

## Abstract (editor highlights)

Publisher’s note: Undertaking any experimental protocol requires adherence to local institutional guidelines for laboratory safety and ethics.

## Graphical abstract

**Figure:**
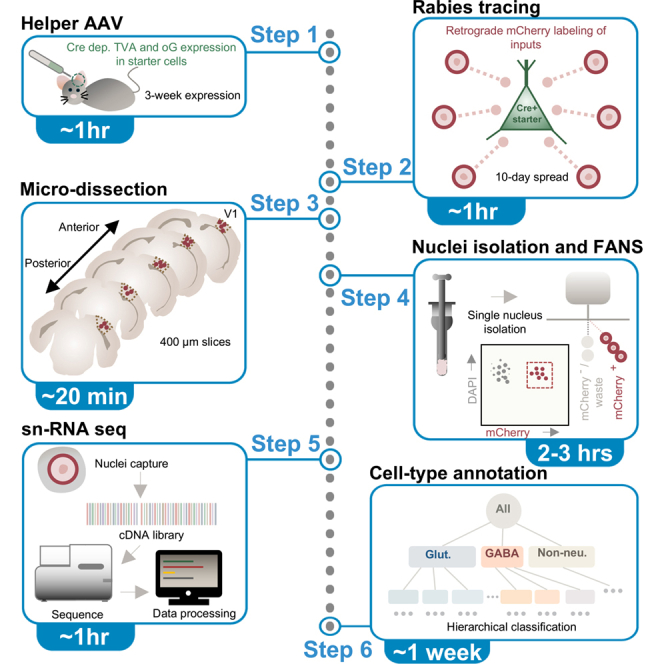

## Before you begin

The protocol below describes START applied to map local cortical inputs to layer-specific excitatory neurons in mouse primary visual cortex (V1) using different Cre-driver mouse lines (Sepw1-Cre for L2/3, Scnn1a-Cre for L4, Tlx3-Cre for L5 IT, Npr3-Cre for L5 ET, and Ntsr1-Cre for L6 CT neurons). However, this approach is generalizable to any genetically defined neuronal population and brain region of interest. The key requirements are: (1) a Cre-driver line that labels your starter population, (2) stereotaxic coordinates for your target region, and (3) appropriate timing considerations for viral expression based on your specific application.

### Innovation

Single transcriptome-assisted rabies tracing (START) integrates three established techniques (monosynaptic rabies tracing, fluorescence-activated nuclei sorting, and single-nuclei RNA sequencing) into a unified workflow that enables transcriptomic identification of presynaptic input neurons at subtype resolution. While monosynaptic rabies tracing has been widely used to map circuit connectivity and single-cell transcriptomics to classify neuronal cell types, prior approaches have not enabled direct linkage between monosynaptic connectivity and transcriptomic identity at scale. The key innovation of START is the coupling of genetically restricted rabies-based circuit tracing with nuclei-based transcriptomic profiling of rabies-labeled input neurons. This strategy allows presynaptic neurons to be classified according to established transcriptomic taxonomies rather than inferred indirectly from anatomical location, morphology, or marker expression. The use of single-nuclei RNA sequencing enables robust transcriptomic profiling of rabies-infected neurons while preserving compatibility with viral tracing and fluorescence-based sorting. START incorporates supervised label transfer from a high-quality reference dataset to assign rabies-labeled neurons to classes, subclasses, and transcriptomic subtypes. START resolves cortical connectivity motifs at a level of cell-type granularity that is not achievable using existing tracing or genetic targeting approaches alone. The protocol is modular and adaptable, enabling its application to diverse genetically defined neuronal populations and brain regions.

### Institutional permissions

All animal experiments described in this protocol were approved by the Salk Institute Institutional Animal Care and Use Committee (IACUC) and conform to the NIH guidelines for the care and use of laboratory animals. Researchers adopting this protocol must obtain approval from their local IACUC or equivalent regulatory body before beginning experiments. Additional Institutional Biosafety Committee approval is required for work with rabies virus. Researchers must verify that their institution has appropriate Biosafety Level 2 (BSL-2) facilities and that all personnel are trained in the safe handling of these viral agents. All animal procedures, housing requirements, and biosafety protocols must adhere strictly to local and national regulatory standards.

### Prepare viral stocks

**Timing: 15 min**

1. Thaw AAV8-DIO-TC66T-2A-oG helper virus on ice.

***Note:*** Aliquot viruses in single-use volumes in sterile Eppendorf tubes on the day of the experiment to avoid repeated freeze-thaw cycles, which reduce viral titer. Store at −80°C until the day of injection.

2. Dilute the helper AAV in sterile 1× PBS (pH 7.4) to achieve final titer of approximately 4 × 10^12^ GC/mL.
  a. Mix 2 μL AAV stock with 10 μL PBS for 12 μL total volume.
  b. Mix gently by slowly pipetting up and down 5–10 times. .

***Note:*** Keep on ice until injection.

3. Thaw EnvA^+^RVdG-H2B-mCherry aliquot on ice.

***Note:*** Use undiluted at stock titer (approximately 7 × 10^7^ IU/mL). Keep on ice until injection

### Pull glass injection pipettes

**Timing: 30 min (for batch of 10 pipettes)**

4. Prepare storage dish by placing a strip of dental wax along the bottom of a large Petri dish.
5. Pull borosilicate glass micropipettes using a pipette puller (e.g., Sutter P-97) to produce long, tapered pipettes.
  a. Example settings in Sutter P-97: HEAT RAMP+20, TIME 44, VEL 64, PUL 64.

***Note:*** Settings will vary by puller model, glass type, and capillary dimensions.

6. Under a dissection microscope, use fine forceps to break pipette tips to achieve an approximately 30 μm tip diameter.
7. Stick pulled pipettes into the dental wax in the petri dish for storage.
8. Cover the Petri dish and store in a dust-free location until use.

***Note:*** Pipettes can be pulled weeks in advance and stored in a dust-free container.

### Prepare surgical tools and workspace

**Timing: 15–20 min**

9. Gather surgical instruments and materials.
  a. Surgical tools: fine forceps, scalpel with blade, small scissors, clamps or retractors.
  b. Drilling equipment: Drill with 0.7 mm drill bits.
  c. Disinfection supplies: Betadine, 70% ethanol, sterile cotton swabs, sterile saline.
  d. Anesthesia and analgesia: Isoflurane, ophthalmic ointment, Buprenorphine-SR (0.5–1 mg/kg), ibuprofen in drinking water (30 mg/kg).
  e. Wound closure: 5–0 absorbable sutures.
  f. Recovery supplies: Heating pad, recovery cage.
10. Sterilize all reusable surgical instruments using an autoclave.
  a. Allow instruments to cool completely before use.
  b. Store sterile instruments in a covered container until surgery.
11. Prepare sterile surgical workspace.
  a. Clean surgical area and stereotaxic frame with 70% ethanol.
  b. Lay out sterile drape or field for instruments.
  c. Ensure adequate lighting and access to anesthesia delivery system.
12. Set up stereotaxic frame with nose cone attached for continuous isoflurane delivery.
13. Prepare post-operative care area with heating pad and clean cage.

***Note:*** Complete on day of surgery before beginning injections. Maintain aseptic technique throughout surgery to minimize risk of infection.

### Prepare coated PCR collection tubes for FACS

**Timing: 30 min**

14. Add 50 μL of 5% BSA solution to each 0.2 mL PCR tube.
15. Vortex tubes for 10 seconds to coat tube walls thoroughly.
16. Remove BSA solution by pipetting or aspiration.
17. Allow tubes to air dry completely at room temperature (20°C–25°C) (approximately 20–30 min).
18. Once dry, prepare collection buffer by adding to each coated tube:
  a. 1.5 μL BSA (2.5%, diluted from 5% stock).
  b. 1.5 μL RNasin Plus (4 U/μL, diluted 1:10 from stock).
  c. 12 μL 1× PBS (pH 7.4, RNase-free).

***Note:*** Keep coated tubes with collection buffer on ice until FACS sorting.

***Note:*** Prepare on day of FACS sorting. The total collection volume is 15 μL. The added PBS helps cushion nuclei during sorting and mitigates nuclei loss during collection.

## Key resources table

**Table:** 

REAGENT or RESOURCE	SOURCE	IDENTIFIER
**Bacterial and virus strains**		

AAV8-DIO-TC66T-2A-oG	Salk Vector Core GT3	Construct: AAV8-DIO-TC66T-2A-oG; titer 4.31×10^13^ GC/mL
EnvA+RVdG-H2BmCherry	Salk Vector Core GT3	Construct: EnvA+RVdG-H2BmCherry; titer 7.43×10^7^ GC/mL

**Chemicals, peptides, and recombinant proteins**		

DL-Dithiothreitol	Sigma	Cat#646563
Protease inhibitor	Sigma	Cat#P8340
RNasin Plus RNase inhibitor	Promega	Cat#N2611
DAPI (4′,6-Diamidino-2-Phenylindole, Dihydrochloride)	Thermo Fisher	Cat#D1306
Triton X-100	Sigma	Cat#93443
Tris-HCl 1M pH 7.4	Boston Bioproducts	Cat#R-314
MgCl_2_ 1M	Ambion	Cat#AM9530G
KCl 2M	Ambion	Cat#AM9640G
Nuclease-free water	Fisher	Cat#BP561-1
Ultrapure BSA, nuclease-free	Ambion	Cat#AM2616
Sucrose	Sigma-Aldrich	Cat#S0389
Glucose	Sigma-Aldrich	Cat#G8270
CaCl_2_	Sigma-Aldrich	Cat#C1016
NaH_2_PO_4_	Sigma-Aldrich	Cat#S0751
NaHCO_3_	Sigma-Aldrich	Cat#S5761
Paraformaldehyde	Sigma-Aldrich	Cat#P6148

**Critical commercial assays**		

Chromium Next GEM Single Cell 3ʹ Kit v3.1	10× Genomics	PN-1000268
Chromium Next GEM Single Cell 3ʹ Kit v3.1	10× Genomics	PN-1000269
Chromium Next GEM Chip G Single Cell Kit	10× Genomics	PN-1000127
Dual Index Kit TT Set A	10× Genomics	PN-1000215
High Sensitivity D1000 ScreenTape	Agilent Technologies	Cat# 5067-5584
High Sensitivity D1000 Reagents	Agilent Technologies	Cat# 5067-5585

**Deposited data**		

Single-nuclei RNA sequencing dataset (rabies tracing)	Patiño et al.^1^	GEO: GSE261436
Single-nuclei RNA sequencing dataset (reference)	Patiño et al.^2^	GEO: GSE196771

**Experimental models: Organisms/strains**		

Mouse: Sepw1-Cre NP39; *Mus musculus*; C57BL/6J background; male and female; adult (P49–P60)	MMRRC	MMRRC: 036190
Mouse: Scnn1a-Tg3-Cre; *Mus musculus*; C57BL/6J background; male and female; adult (P49–P60)	Jackson Labs	JAX Stock No: 009613
Mouse: Tlx3-Cre PL56*; Mus musculus*; C57BL/6J background; male and female; adult (P49–P60)	MMRRC	MMRRC: 041158
Mouse: Npr3-IRES-Cre-neo; *Mus musculus*; C57BL/6J background; male and female; adult (P49–P60)	Jackson Labs	JAX Stock No: 031333
Mouse: Ntsr1-Cre GN220; *Mus musculus*; C57BL/6J background; male and female; adult (P49–P60)	MMRRC	MMRRC: 030648

**Software and algorithms**		

CellRanger [v6.0]	10× Genomics	RRID:SCR_017344
R [v4.1.1]	The R Project	RRID:SCR_001905
DoubletFinder [v2.0.2]	McGinnis et al.^3^	RRID:SCR_018771
SingleR [v4.1]	Aran et al.^4^	RRID:SCR_023120
Seurat [v4.0.4]	Butler et al.^5^	RRID:SCR_007322

**Other**		

P-97 Micropipette Puller	Sutter Instrument	RRID:SCR_018636
Leica VT1200 vibratome	Leica Biosystems	RRID:SCR_018453
SZX16 dissection microscope	Olympus	RRID:SCR_018782
BX63 microscope	Olympus	RRID:SCR_018543
BD FACS Aria Fusion	BD Biosciences	RRID:SCR_018434
Agilent 4150 TapeStation	Agilent Technologies	RRID:SCR_025082
Chromium controller	10× Genomics	RRID:SCR_019326
NovaSeq™ 6000	Illumina	RRID:SCR_016387
1.5 mL microcentrifuge tubes	Thermo Fisher	Cat#02-681-320
40 μm cell strainer	Corning	Cat#431750
Borosilicate glass capillaries	VWR	Cat#53432-706
Dounce Tissue Grinder Set, 2 mL	DWK Life Sciences	Cat# 885300-0002

## Materials and equipment

**Table:** Concentrated 10× dissection buffer:

Reagent	Final concentration	Amount
KCl	25 mM	1.865 g
CaCl_2_	5 mM	0.735 g
MgCl_2_	70 mM	14.22 g MgCl_2_
NaH_2_PO_4_	12.5 mM	1.725 g
H_2_O	N/A	to 1L
**Total**	**N/A**	**1 L**

Store at 4°C for up to 6 months.

**Table:** Slicing solution: 250 ml per mouse

Reagent	Final concentration	Amount
Sucrose	110 mM	9.41 g
Glucose	10 mM	0.525 g
NaHCO_3_	25 mM	0.525 g
10× Dissection Buffer	1×	25 mL
ddH_2_O	N/A	to 250 mL
**Total**	**N/A**	**250 mL**

Prepare fresh on day of dissection. Filter solution and freeze at −80°C for 40 min to chill rapidly. Continuously bubble with carbogen (95% O_2_/5% CO_2_) for at least 10 min before use and throughout tissue processing. Keep on ice during entire dissection procedure.

**Table:** Nuclei isolation medium (NIM): 2.5 mL per sample

Reagent	Final concentration	Amount
Sucrose 1 M	0.25M	0.625 mL
KCl 2 M	25 mM	31.25 μL
MgCl_2_ 1 M	5 mM	12.5 μL
Tris-HCl (pH 7.4) 1 M	10 mM	25 μL
H_2_O	N/A	1.807 mL
**Total**	**N/A**	**2.5 mL**

Store the NIM base at 4°C for up to 6 months.

**Table:** Nuclei isolation medium with DAPI and protease inhibitors (NIM-DP): 2.5 mL per sample

Reagent	Final concentration	Amount
NIM (from above)	N/A	2.5 mL
DTT 1 M	1 mM	2.5 μL
Protease inhibitor 50×	1×	25 μL
RNasin Plus 40 U/μL	N/A	3.5 μL
**Total**	**N/A**	**2.531 ml**

Prepare fresh on day of nuclei isolation. Do not store. Add 0.1% Triton X-100 (10 μL per 1 mL NIM-DP from 10% stock) and 10 μM DAPI (1 μL per 1 mL NIM-DP from 1 mg/mL stock) immediately before use.

**Table:** Nuclei storage buffer base (NSB): 0.6 mL per sample

Reagent	Final concentration	Amount
Sucrose 1 M	0.25 M	150 μL
MgCl_2_ 1 M	5 mM	3 μL
Tris-HCl (pH 7.4) 1 M	10 mM	6 μL
H_2_O	N/A	0.440 mL
**Total**	**N/A**	**0.6 mL**

Store NSB base at 4°C for up to 6 months.

**Table:** Nuclei storage buffer with protease inhibitors (NSB-DP): 0.6 mL per sample

Reagent	Final concentration	Amount
NSB (from above)	N/A	0.6 mL
DTT 1 M	1 mM	0.6 μL
Protease inhibitor 50×	1×	12 μL
RNasin Plus 40 U/μL	N/A	0.9 μL
**Total**	**N/A**	**0.6135 ml**

Prepare fresh on day of nuclei isolation. Do not store.

## Step-by-step method details

### Cre-dependent helper AAV injection for starter cell labeling

**Timing: 45–60 min per mouse; wait period: 3 weeks**

**Figure 1:**
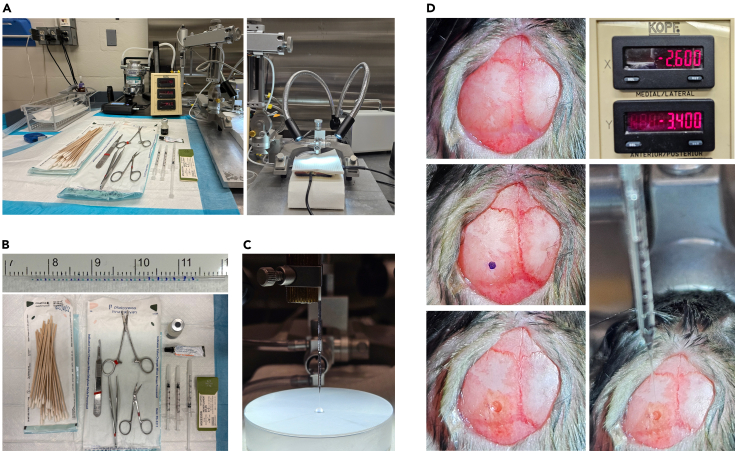
Stereotaxic injection setup for viral delivery to primary visual cortex (V1) (A) Left: Overview of the surgical workspace including the isoflurane anesthesia system, stereotaxic frame, surgical instruments, and Kopf digital coordinate readout. Right: Close-up of the stereotaxic frame showing nose cone, heating pad, and fiber optic illumination. (B) Top: Representative pulled glass pipettes used for viral delivery. Bottom: Surgical instruments and materials used for the craniotomy procedure. (C) Backfilling of the pulled glass pipette with viral solution prior to injection. (D) Representative surgical images showing the exposed skull following midline incision and skin retraction, with greater exposure on the left hemisphere for injection. The target V1 coordinates (2.6 mm lateral, 3.4 mm posterior to bregma) are marked with a dot on the skull surface. Subsequent images show the completed craniotomy and the pulled glass pipette positioned over the craniotomy site for injection.

This step establishes the starter cell population by injecting AAV8-DIO-TC66T-2A-oG into genetically defined neurons (Cre-positive cells) to express TVA receptor and rabies glycoprotein. These components are essential for subsequent rabies infection and retrograde tracing (Figure 1).

1. Back-fill pulled glass pipette with diluted AAV helper virus.
  a. Mount pulled glass pipette onto stereotaxic holder.
  b. Connect pipette to a 1 mL syringe via an 18G tubing adaptor and tubing.
  c. Place the tip of mounted pipette into diluted AAV helper virus solution.
  d. Gently pull back on the syringe plunger to draw virus solution into pipette. Fill pipette with sufficient volume for injection (approximately 100–200 nL).
  e. Expel a small drop of solution from pipette tip to ensure no clogs or trapped air in the system.

***Note:*** Mineral oil may be necessary if a plunger-based or oil-hydraulic injection system (e.g., Nanoject, Wiretrol) is used as an alternative. In those cases, follow the manufacturer's instructions for backfilling with mineral oil.

2. Place mouse in induction chamber.
  a. Start isoflurane flow at 2% and set the oxygen flow meter to 1–2 L/min rate.
  b. Assess loss of consciousness of the mouse via toe pinch.
  c. Transfer the mouse to the surgical surface.
3. Place mouse on a heating pad to maintain body temperature throughout surgery.
4. Switch the anesthesia tubing to the nose cone.
  a. Place the mouse’s nose in the nose cone and decrease isoflurane concentration to 1.5%.
  b. Apply ophthalmic ointment to eyes to prevent drying.
5. Administer Buprenorphine-SR (0.5–1 mg/kg, subcutaneous) for analgesia.
6. Position the mouse in the stereotaxic frame by adjusting the ear bars.
  a. Make sure to not damage eardrums and that there are no signs of head movement.
  b. Administer 1–2 mg (0.05–0.1 mL) of 2% lidocaine subcutaneously at the location of the planned scalp incision.
  c. Clean the head of the mouse with alternating Betadine and 70% ethanol scrubs (3 cycles).
  d. Make a rostro-caudal incision (∼1 cm) on the scalp along the midline using a scalpel blade.
  e. Use a pair of clamps to retract skin and expose the skull.
  f. Clean the skull with sterile saline and dry with a cotton swab.
7. Calibrate the stereotaxic coordinate system.
  a. Find the bregma position. Lower the pipette tip until it touches the skull.
  b. Reset all the stereotaxic coordinates to zero at bregma.
  c. Retract the pipette from the skull.
  d. Move the pipette to lambda and lower it until it touches the skull.
  e. Check that the DV value is 0 ± 0.01 mm (indicating a level skull).

**CRITICAL:** If the DV value exceeds ±0.01 mm, adjust ear bars or head position to level the skull before proceeding.

8. Retract the pipette from the skull once the system is calibrated and move the stereotaxic arm to the target coordinates for V1: 3.4 mm posterior and 2.6 mm lateral relative to bregma.
9. Create small craniotomy (∼0.5 mm diameter) at target coordinates using a drill.
  a. Drill carefully to avoid penetrating dura mater.
  b. Remove bone fragments with fine forceps.
10. Slowly lower pipette through craniotomy to target depth: 0.5–0.7 mm ventral from pia (targeting center of V1).
11. Wait 2 min after reaching target depth to allow tissue to settle.
12. Inject 100 nL of diluted AAV at rate of approximately 20 nL/min using gentle, steady manual pressure on syringe plunger.
  a. Monitor injection by observing meniscus movement in pipette.

***Note:*** Total injection time should be approximately 5 min.

13. Wait 5 min post-injection with pipette in place to prevent backflow along injection tract.

**CRITICAL:** The 5-min waiting period is essential to prevent viral solution from being pulled back along the injection tract when the pipette is withdrawn.

14. Slowly withdraw pipette from brain.
15. Close scalp incision using 5–0 absorbable sutures.
16. Remove mouse from stereotaxic frame and place it in recovery cage on heating pad.
  a. Monitor until mouse is ambulatory.
17. Provide ibuprofen in drinking water (30 mg/kg) for 48 h post-surgery for analgesia.
18. Wait 3 weeks to allow AAV expression of TVA receptor and rabies glycoprotein in starter cells.

**CRITICAL:** The 3-week interval between helper AAV injection and rabies injection is essential for robust expression of TVA receptor and glycoprotein in Cre-positive starter cells.

**Pause point:** Animals are housed for 21 days before rabies injection.

### EnvA-pseudotyped rabies virus injection

**Timing: 45–60 min per mouse; wait period: 10 days**

Three weeks after helper AAV injection, inject EnvA-RVdG-H2B-mCherry at the same V1 coordinates to initiate retrograde tracing from starter cells. The rabies virus will infect only TVA-expressing starter cells and spread retrogradely to monosynaptically connected input neurons.

19. Back-fill pulled glass pipette with EnvA-RVdG-H2B-mCherry.
  a. Mount pulled glass pipette onto stereotaxic holder.
  b. Connect pipette to 1 mL syringe via an 18G tubing adaptor and tubing.
  c. Place tip of mounted pipette into undiluted rabies virus solution.
  d. Gently pull back on syringe plunger to draw virus solution into pipette.
  e. Fill pipette with sufficient volume for injection (approximately 200–300 nL).
  f. Expel a small drop of solution from pipette tip to ensure no clogs or trapped air in the system.

**CRITICAL:** Handle rabies virus only in BSL-2 conditions with appropriate personal protective equipment. Follow institutional biosafety protocols at all times.

20. Place mouse in induction chamber.
  a. Start isoflurane flow at 2% and set the oxygen flow meter to 1–2 L/min rate.
  b. Assess loss of consciousness of the mouse via toe pinch.
  c. Transfer the mouse to the surgical surface.
21. Place mouse on a heating pad to maintain body temperature throughout surgery.
22. Switch the anesthesia tubing to the nose cone.
  a. Place the mouse’s nose in the nose cone and decrease isoflurane concentration to 1.5%.
  b. Apply ophthalmic ointment to eyes to prevent drying.
23. Administer Buprenorphine-SR (0.5–1 mg/kg, subcutaneous) for analgesia.
24. Position the mouse in the stereotaxic frame by adjusting the ear bars.
  a. Make sure to not damage eardrums and that there are no signs of head movement.
  b. Administer 1 to 2 mg (0.05–0.1 mL) of 2% lidocaine subcutaneously at the location of the planned scalp incision.
  c. Clean the head of the mouse with alternating Betadine and 70% ethanol scrubs (3 cycles).
  d. Make a rostro-caudal incision (∼1 cm) on the scalp along the midline using a scalpel blade.
  e. Use a pair of clamps to retract skin and expose the skull.
  f. Clean the skull with sterile saline and dry with a cotton swab.
25. Calibrate the stereotaxic coordinate system.
  a. Find the bregma position. Lower the pipette tip until it touches the skull.
  b. Reset all the stereotaxic coordinates to zero at bregma.
  c. Retract the pipette from the skull.
  d. Move the pipette to lambda and lower the pipette until it touches the skull.
  e. Check that the DV value is 0 ± 0.01 mm (indicating a level skull).
  f. Retract the pipette from the skull once the system is calibrated and move the stereotaxic arm to the same target coordinates used for AAV injection: 3.4 mm posterior and 2.6 mm lateral relative to bregma.

**CRITICAL:** It is critical to inject rabies at the same coordinates as the helper AAV to ensure maximal overlap with starter cells.

26. Locate the previous craniotomy site from AAV injection.
  a. Identify the craniotomy on the skull surface.
  b. If scar tissue has formed over the craniotomy, carefully remove it using fine forceps.
  c. Ensure the opening is clear for pipette entry.

***Note:*** The craniotomy is typically still visible at this stage; if not, the scalp landmarks and stereotaxic coordinates from the AAV injection can serve as the primary guide.

27. Slowly lower pipette through craniotomy to the same target depth used for AAV injection: 0.5–0.7 mm ventral from pia.
28. Wait 2 min after reaching target depth to allow tissue to settle.
29. Inject 200 nL of rabies virus at a rate of approximately 20 nL/min using gentle, steady manual pressure on the syringe plunger.
  a. Monitor injection by observing meniscus movement in the pipette.

***Note:*** Total injection time should be approximately 10 min.

30. Wait 5 min post-injection with pipette in place to prevent backflow along injection tract.
31. Slowly withdraw pipette from brain over 1 to 2 min.
32. Close scalp incision using 5–0 absorbable sutures.
33. Remove mouse from stereotaxic frame and place in recovery cage on heating pad.
  a. Monitor continuously until mouse is ambulatory.
34. Provide ibuprofen in drinking water (30 mg/kg) for 48 h post-surgery for analgesia.
35. House the mouse under BSL-2 conditions for 10 days to allow rabies retrograde spread and mCherry expression in input neurons.

**CRITICAL:** The 10-day expression period is optimized to maximize labeled input neurons while maintaining cell viability.

**Pause point:** Animals are housed for 10 days post-rabies injection before sacrifice.

### Brain tissue dissection and V1 microdissection

**Timing: 20–30 min per mouse**

**Figure 2:**
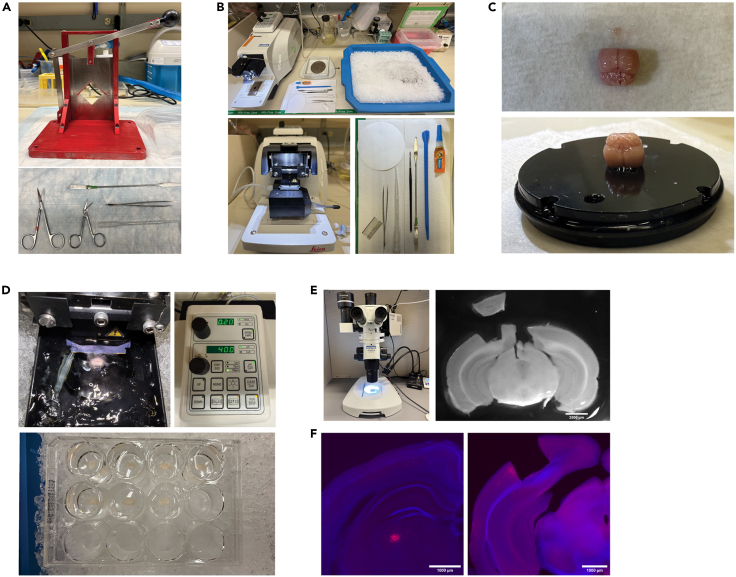
Brain dissection and V1 micro-dissection workflow (A) Small animal guillotine for decapitation, with dissection tools for brain harvesting: fine forceps, angled scissors, surgical scissors, double-ended spatula, and a plastic transfer pipette for applying ice-cold slicing solution to the harvested brain. (B) Leica VT1200 vibratome setup and tools needed for sectioning. Workstation showing vibratome instrument, ice bucket, 12-well collection plate, and mounting tools including super glue for mounting the brain onto the specimen chuck, a disposable spatula for transferring the brain from the slicing buffer beaker to filter paper, a double-ended spatula, a fine paintbrush, a plastic transfer pipette, fine forceps, and a blade for trimming the anterior portion of the brain to create a flat mounting surface. (C) Example of how to mount brain on vibratome holder. Anterior portion of brain trimmed to create a flat mounting surface. Brain positioned on specimen holder and secured for stable sectioning. (D) Top left: Brain mounted on vibratome in slicing chamber. Top right: Vibratome parameters displayed. Bottom: Coronal sections collected into 12-well plate containing ice-cold dissection buffer. (E) Micro-dissection of V1 region. Left: Dissection microscope (Olympus SZX16) setup. Right: Representative coronal section after V1 micro-dissection showing excised V1 tissue adjacent to the remaining brain slice. Scale bar = 2500 um. (F) Post-hoc validation of accurate micro-dissection. Remaining brain sections (after V1 has been removed for nuclei isolation) can be fixed in 4% PFA, stained with DAPI, and imaged to confirm accurate V1 dissection and validate rabies spread. Left: Anterior section showing retrograde spread to lateral geniculate nucleus (LGN). Right: Section after V1 micro-dissection showing absence of dissected V1 region, with labeling visible in lateral higher visual areas, confirming both accurate V1 removal and retrograde rabies spread to connected cortical regions. Scale bars = 1000 um.

Ten days after rabies injection, harvest brain tissue and micro-dissect V1 regions containing mCherry-positive rabies-labeled neurons for subsequent nuclei isolation (Figure 2).

36. Prepare slicing solution according to recipe in Materials and Equipment section (250 mL per mouse).
  a. Filter solution into 500 mL collection bottle.
  b. Freeze at −80°C for 40 min to chill rapidly.

***Note:*** For multiple mice, scale up slicing solution accordingly.

37. Transfer ice-cold slicing solution to beaker and oxygenate with carbogen (95% O_2_/5% CO_2_) for 10 min prior to brain removal.

***Note:*** Keep solution on ice throughout. Maintain continuous carbogen flow during entire tissue processing.

38. Prepare workspace and collection vessels.
  a. Assemble vibratome by placing the slicing chamber (filled with carbogen-bubbled slicing buffer) containing mounted brain specimen into ice-packed outer tray.
  b. Prepare 12-well plates with dissection buffer for slice collection.
39. Deeply anesthetize mouse with isoflurane, then euthanize by decapitation.
  a. Place mouse in induction chamber with 5% isoflurane.
  b. Confirm deep anesthesia and lack of response to toe pinch.
  c. Proceed immediately to decapitation with small animal guillotine or large scissors.
40. Rapidly extract brain and immediately submerge in ice-cold, oxygenated slicing solution.
41. Allow brain to equilibrate in cold slicing solution for 10 min on ice.
42. Prepare brain for mounting.
  a. Trim anterior portion of brain with a blade to create a flat mounting surface.
  b. Apply super glue to surface of specimen chuck.
  c. Mount brain onto chuck with flattened anterior surface adhered to the glue and posterior side oriented upward.
43. Set vibratome parameters: 400 μm section thickness, 0.20 mm/s blade advance speed, 100 μm step size.
44. Section brain coronally at 400 μm thickness.
  a. Collect sections sequentially in 12-well plates containing ice-cold slicing solution.

***Note:*** Keep sections on ice and oxygenated.

45. Place collected slices at 4°C until ready for micro-dissection.
46. Visualize V1 regions using a dissection microscope (Olympus SZX16).

***Note:*** V1 can be identified using anatomical landmarks or by visualizing mCherry fluorescence with appropriate filter set (Excitation: 587/25 nm, Emission: 610 nm long-pass).

47. Micro-dissect V1 regions using fine forceps and microscissors.
48. Transfer dissected V1 tissue immediately to a pre-chilled 1.5 mL microcentrifuge tube.
49. Immediately freeze the tissue on dry ice.

***Note:*** Although direct nuclear extraction from fresh tissue is also feasible for snRNA-seq, this protocol uses snap-frozen tissue to allow banking and balanced batch-processing of samples across experimental conditions.

50. Store frozen tissue at −80°C until nuclei isolation.
51. Fix the remaining brain slices in 4% paraformaldehyde (PFA) for 12–16 hat 4°C for post-hoc validation.
  a. After fixation, stain with DAPI.
  b. Image the tissue to validate accurate V1 dissection and document rabies spread to connected regions including lateral geniculate nucleus (LGN) and higher visual areas.

***Note:*** PFA-fixed brain slices can be cryopreserved for long-term storage by transferring to 30% sucrose in PBS at 4°C until the slices sink, followed by storage at −20°C. This may be useful for users wishing to bank fixed tissue for later histological analysis.

**Pause point:** Frozen tissue can be stored at −80°C for up to 6 months before nuclei isolation.

### Single-nucleus isolation and fluorescence-activated nuclei sorting

**Timing: 2–3 h**

**Figure 3:**
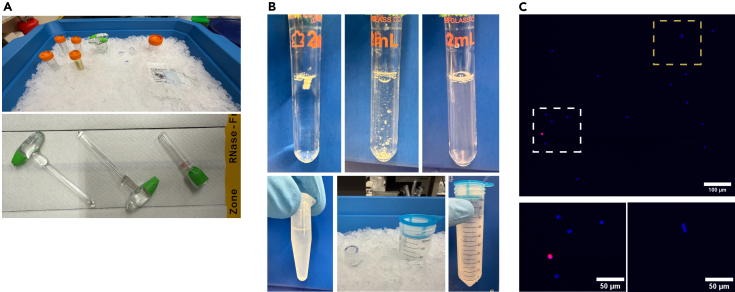
Nuclei isolation from frozen V1 tissue (A) Nuclei isolation setup. Dounce homogenizer on ice with pre-chilled tubes and buffers for nuclei extraction from frozen V1 tissue (B). (C) Quality assessment of isolated nuclei. Top: Representative field showing isolated nuclei with DAPI (blue) and mCherry fluorescence (red). Low proportion of mCherry+ rabies-labeled nuclei (white dashed box) visible prior to FACS sorting and minimal clumping visible (yellow dashed box), indicating successful isolation with maintained nuclear integrity suitable for FACS sorting. Scale bar = 100 um. Bottom left: Enlarged view of white dashed box region from top panel showing mCherry+ rabies-labeled nuclei (red). Scale bar = 50 um. Bottom right: Enlarged view of yellow dashed region from top panel showing two clumped nuclei, which should be rare in a properly prepared sample. Scale bar = 50 um.

**Figure 4:**
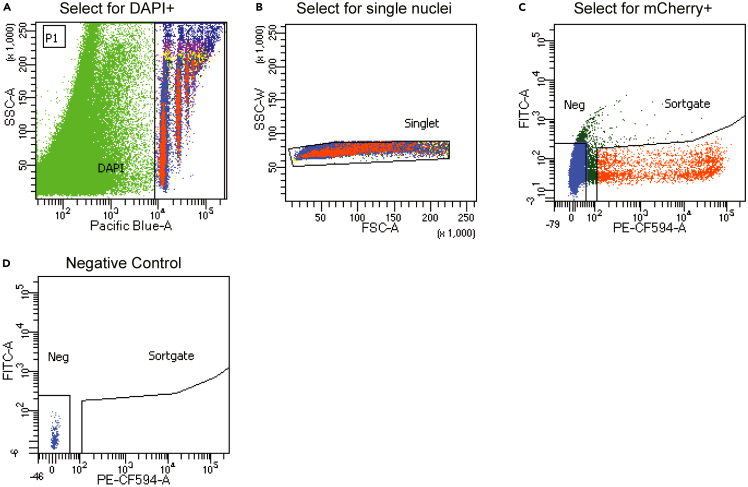
FACS gating strategy (A–D) Detailed gating strategy for FACS sorting of mCherry^+^ rabies-labeled nuclei. (A) Gate selects for DAPI^+^ nuclei to exclude debris. (B) Gate to exclude doublet nuclei based on single nuclei morphology. (C) Gate to select for mCherry^+^ fluorescence. (D) A negative control sample from uninfected tissue used to set mCherry-negative gate.

Extract nuclei from frozen tissue and sort mCherry-positive, rabies-labeled nuclei (Figures 3 and 4).

52. Thaw reagents on ice.
  a. DTT, sucrose, KCl, MgCl_2_, Tris-HCl stocks on ice.
  b. Keep the protease inhibitor at room temperature (20°C–25°C).
53. Turn on centrifuge and set to 4°C to cool.
54. Pre-chill labware on ice.
  a. 1.5 mL microcentrifuge tubes.
  b. 50 mL conical tubes.
  c. 40 μm cell strainers.
55. Prepare NIM-DP (nuclei isolation medium with DAPI and protease inhibitors) according to the recipe in the materials and equipment section.
  a. Prepare approximately 2 mL NIM-DP per sample.

***Note:*** Keep on ice.

56. Prepare NSB-DP (nuclei storage buffer with protease inhibitors) according to the recipe in the materials and equipment section.
  a. Prepare approximately 500 μL NSB-DP per sample.

***Note:*** Keep on ice.

57. Pre-chill dounce homogenizer and pestle on ice.
  a. Place dounce homogenizer and pestle in 15 mL tube to avoid contamination.
  b. Chill for at least 10 min.
58. Add 900 μL NIM-DP and 9 μL of 10% Triton X-100 to a pre-chilled dounce homogenizer.
59. Chill dounce with NIM-DP/Triton mixture on ice for 10 min.
60. Retrieve frozen tissue sample from −80°C freezer and place on dry ice.
61. Carefully transfer tissue to 1 mL dounce homogenizer containing ice-cold NIM-DP/Triton mixture.
62. Homogenize tissue with loose pestle using 5 gentle strokes.

***Note:*** Work on ice throughout and avoid introducing air bubbles.

63. Add 1 μL DAPI (1 mg/mL stock) to homogenate.
64. Continue homogenization with tight pestle using 15 strokes.

***Note:*** Maintain gentle, steady strokes, avoid introducing air bubbles, and keep homogenizer on ice throughout.

65. Transfer homogenate to pre-chilled 1.5 mL microcentrifuge tube.
66. Centrifuge at 1,000 × *g* for 8 min at 4°C to pellet nuclei.
67. Carefully aspirate supernatant without disturbing the pellet.

***Note:*** Leave approximately 100 μL in tube to avoid losing the pellet.

68. Gently resuspend pellet in 1 mL NIM-DP by pipetting up and down 10–15 times.

***Note:*** Avoid vigorous pipetting, which can damage nuclei.

69. Centrifuge at 1,000 × *g* for 8 min at 4°C.
70. Carefully aspirate supernatant without disturbing pellet.

***Note:*** Leave approximately 100 μL in tube to avoid losing pellet.

71. Gently resuspend pellet in 450 μL NSB-DP by pipetting up and down 10–15 times.
72. Filter nuclei suspension through a pre-chilled 40 μm cell strainer into 50 mL conical tube.

***Note:*** Keep strainer and collection tube on ice.

73. Add 50 μL nuclease-free BSA (final concentration 1%) to the filtered nuclei suspension.
74. Mix gently and wrap sample tube with aluminum foil to protect from light.
75. Place sample on rotator or shaker at 4°C until ready for FACS sorting.

***Note:*** Proceed to FACS sorting as soon as possible.

76. Transfer nuclei suspension to FACS sorting tube.
77. Transport pre-chilled coated collection tubes (prepared in the before you begin section) to FACS core facility on ice.
78. Set up FACSAria Fusion sorter with a 70 μm nozzle at 22.5 PSI sheath pressure.
  a. Use Pacific Blue channel (Excitation: 405 nm) for DAPI.
  b. Use PE-Texas Red channel (Excitation: 561 nm) for mCherry.
79. Process uninfected control nuclei to set the negative FANS gate for mCherry.
  a. Use nuclei from a non-injected mouse processed identically.
  b. Gate on DAPI-positive events to identify nuclei.
  c. Set mCherry-negative gate based on the control sample.
80. Sort DAPI^+^/mCherry^+^ rabies-labeled nuclei into pre-chilled, coated PCR collection tubes.

***Note:*** Target 5,000–10,000 nuclei per sample.

81. Immediately load sorted nuclei onto 10× Genomics Chromium Controller.

### 10× Genomics single-nucleus RNA sequencing

**Timing: 2 days for library prep; 1 day for sequencing**

**Figure 5:**
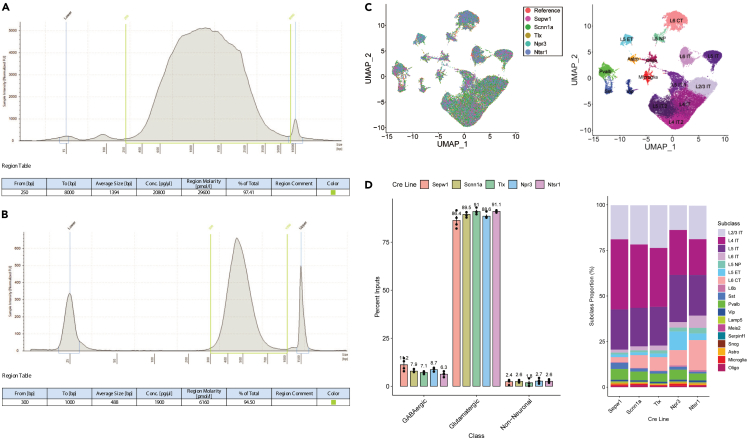
10× Genomics library quality control and transcriptomic cell-type annotation (A and B) Representative Bioanalyzer traces showing quality of cDNA and final sequencing libraries. (A) cDNA quality assessment showing expected size distribution of 250–8,000 bp. (B) Final library quality showing characteristic peak around 500 bp with size range of 300–1000 bp, indicating successful library preparation and readiness for sequencing. (C) Uniform Manifold Approximation and Projection (UMAP) of rabies-labeled nuclei color coded by the Cre-line tracing experiment they were collected from (left) or by subclass identity (right). (D) Left: Proportions of GABAergic, glutamatergic, and nonneuronal nuclei passing QC across Cre-driver mouse lines. Right: Proportion of distinct subclasses across Cre-driver mouse lines. (C and D) Reprinted with permission from Patiño et al.^1^

Generate single-nucleus cDNA libraries from sorted rabies-labeled nuclei using the 10× Genomics Chromium platform and sequence on Illumina NovaSeq to obtain transcriptomic profiles of input neurons (Figures 5A and 5B).

82. Prepare single-nucleus capture and barcoding using Chromium Next GEM Single Cell 3′ Kit v3.1 Dual Index (10× Genomics, PN-1000268 and PN-1000127).
  a. Use Chromium Next GEM Chip G (PN-1000127) for nuclei capture.

***Note:*** For detailed instructions, refer to the Chromium Next GEM Single Cell 3′ Reagent Kits v3.1 (Dual Index) User Guide (CG000315 Rev F).

83. Load sorted nuclei onto 10× Genomics Chromium Controller.

***Note:*** Target 6,000–10,000 sorted nuclei per channel. Expected recovery: 5,000–7,000 nuclei after doublet removal.

***Note:*** For detailed instructions, refer to the Chromium Next GEM Single Cell 3′ Reagent Kits v3.1 (Dual Index) User Guide (CG000315 Rev F).

84. Process nuclei using Chromium Next GEM Single Cell 3′ Kit v3.1 Dual Index (10× Genomics, PN-1000268 and PN-1000127).
  a. Perform single-nucleus capture and barcoding.
  b. Complete reverse transcription (53°C for 45 min).
  c. Amplify cDNA using 12–14 PCR cycles (adjust based on nuclei input).
  d. Construct sequencing libraries with Dual Index Kit TT Set A (PN-1000215).

***Note:*** For detailed instructions, refer to the Chromium Next GEM Single Cell 3′ Reagent Kits v3.1 (Dual Index) User Guide (CG000315 Rev F).

85. Assess library quality using Agilent Tapestation High Sensitivity DNA Kit.

***Note:*** Expected library size: 400–700 bp with peak around 500 bp.

86. Sequence pooled libraries on Illumina NovaSeq 6000 System (S4 flow cell).
  a. Target sequencing depth: approximately 100,000 reads per nucleus.
  b. Read configuration: Read 1 (28 bp), i7 index (10 bp), i5 index (10 bp), Read 2 (90 bp).

### Computational analysis and transcriptomic cell-type annotation

**Timing: 1–2 weeks**

Process raw sequencing data through alignment, quality control, doublet removal, and supervised cell-type annotation using a reference transcriptomic atlas to identify the transcriptomic subtypes of rabies-labeled input neurons (Figures 5C and 5D).

87. Process raw sequencing data (FASTQ files) using the Cell Ranger pipeline (version 6.0 or later).
  a. Run cellranger count command with mouse pre-mRNA reference transcriptome (mm10).
  b. Include --include-introns flag to incorporate intronic reads in expression quantification.

***Note:*** Cell Ranger outputs feature-barcode matrices containing gene expression counts for each cell barcode. The --include-introns parameter is essential for single-nucleus RNA sequencing as nuclei contain unspliced pre-mRNA.

88. Load Cell Ranger output feature-barcode matrices into R (v4.1.1 or later) using Seurat package (v4.0 or later).

***Note:*** Other single-cell RNA sequencing analysis packages such as Scanpy (Python), Bioconductor SingleCellExperiment, etc., can be used as alternatives to Seurat for data loading and processing.

89. Calculate percentage of mitochondrial transcripts for each nucleus.
  a. Add as metadata to Seurat object using PercentageFeatureSet function.
90. Apply quality control filters to exclude low-quality nuclei.
  a. Remove nuclei with <500 genes detected.
  b. Remove nuclei with >10,000 genes detected (likely doublets).
  c. Remove nuclei with >5% mitochondrial UMIs.

***Note:*** These thresholds are optimized for mouse cortical nuclei and may require adjustment for other brain regions or species.

91. Identify and remove doublets using DoubletFinder package (v2.0 or later) with default parameters.

***Note:*** Doublets represent two nuclei captured in a single droplet. This removal improves accuracy of downstream cell-type annotation.Alternative doublet detection packages include Scrublet, scDblFinder, or DoubletDecon.

92. Download the Allen Institute Brain Science (AIBS) reference single-nucleus RNA sequencing dataset from primary visual cortex.
  a. Access data from GEO: GSE196771.

***Note:*** This protocol uses Allen Institute V1 reference atlas (GEO: GSE196771). For other brain regions, use appropriate regional atlases. Match sequencing technology (10× v3 snRNA-seq) when possible for best results.

93. Perform supervised label transfer from reference dataset to rabies-labeled input neurons using SingleR package (v4.1 or later).
  a. Compute Spearman correlation between each rabies-labeled neuron’s expression profile and each pseudo-bulk reference sample.

***Note:*** SingleR iteratively refines assignments, using only top-scoring cell types from previous iteration until a single annotation remains. At each iteration, only variable genes between top cell types are used to differentiate closely related subtypes. Alternative supervised label transfer methods such as Seurat label transfer function, scmap, or CHETAH can be used instead of SingleR. The key requirement is using a high-quality reference atlas with established cell-type taxonomy.

94. Annotate rabies-labeled neurons at three hierarchical levels.
  a. Class level: Glutamatergic, GABAergic, or non-neuronal.
  b. Subclass level: e.g., L2/3 IT, L4 IT, Pvalb, Sst, Vip, Lamp5.
  c. Subtype level: e.g., Pvalb Vipr2, Sst Chodl, Vip Parm1.
95. Assess annotation confidence using per-cell delta scores.

***Note:*** Delta score = difference between the assigned label score and the median score across all labels. High delta scores indicate confident assignments. Low delta scores indicate uncertain or ambiguous assignments.

96. Exclude low-confidence assignments from downstream analysis.
  a. Remove nuclei with delta scores that are outliers compared to other cells assigned to the same label.

***Note:*** This ensures only high-quality annotations are retained.

97. Classify rare cell subtypes and exclude from statistical analysis.
  a. Define rare subtypes as those with <7 cells assigned in the reference dataset. Note: This corresponds to <0.5% of all inhibitory neurons.
  b. Exclude rare subtypes from downstream statistical comparisons to avoid false positives.
98. Calculate proportions of each transcriptomic subtype among rabies-labeled input neurons for each Cre-driver line.
99. Perform Monte Carlo resampling from reference dataset to establish expected subtype proportions.
  a. For each Cre-driver line, randomly sample n nuclei from reference dataset, where n = the average number of input nuclei obtained across mice from that line.
  b. Perform 10,000 iterations of random sampling.
  c. Calculate mean proportion of each subtype across all iterations as a measure of expected prevalence.
100. Compare experimental input proportions to reference-derived expected proportions.
  a. Use Wilcoxon rank-sum test with Benjamini-Hochberg correction for multiple comparisons.

## Expected outcomes

Successful helper AAV injection should result in robust Cre-dependent expression of TVA receptor and rabies glycoprotein in starter cells within 3 weeks. Subsequent rabies injection at the same coordinates, followed by a 10-days incubation period, should yield widespread mCherry labeling of monosynaptically connected input neurons visible throughout V1 and potentially extending to other cortical and subcortical regions depending on the starter population. The success of rabies tracing can be assessed during tissue dissection (see Tissue dissection and nuclei isolation below) by visualizing mCherry-positive regions under a fluorescent dissection microscope. Successful experiments should show clear mCherry fluorescence in V1 sections, with labeled nuclei distributed throughout the tissue. Additionally, pilot validation experiments can be performed where a subset of mice are perfused and fixed for histological analysis rather than being processed for nuclei isolation. Fixed brain sections should demonstrate accurate targeting of the injection site and appropriate spread of labeled input neurons (Figure 2). This validation step is recommended when establishing the protocol with a new Cre line or a new brain region to confirm successful viral expression and rabies spread.

Brain sectioning on a Vibratome should yield clean 400 μm coronal sections with visible mCherry fluorescence under the dissection microscope (Figure 2). Successful micro-dissection of V1 regions containing rabies-labeled neurons should provide sufficient tissue for downstream nuclei isolation. The nuclei isolation procedure should produce a homogeneous nuclei suspension without visible clumps after filtration through the 40 μm strainer. Note: Pilot experiments can be performed to validate enrichment of mCherry-positive nuclei by imaging a small aliquot of nuclei suspension both pre- and post-FACS sorting under a fluorescent microscope. Pre-sort samples should show a mixture of mCherry-positive and mCherry-negative nuclei, while post-sort samples should be highly enriched for mCherry-positive nuclei (>90% purity). These pilot aliquots would not be used for sequencing but serve to validate the FACS gating strategy and sorting efficiency.

Fluorescence-activated nuclei sorting should yield distinct populations when gating on DAPI and mCherry channels (Figure 4). The DAPI-positive population represents all nuclei, while the DAPI^+^/mCherry^+^ double-positive population represents rabies-labeled input neurons. Using uninfected control tissue to set the mCherry-negative gate is critical for accurate sorting. Typical experiments recover 5,000–10,000 rabies-labeled nuclei per sample, though yields vary depending on the starter population and efficiency of rabies spread. The ratio of inhibitory to excitatory input neurons typically ranges from 6%–11% depending on the starter cell population, consistent with known cortical connectivity patterns.

Library preparation using the 10× Genomics platform should produce high-quality cDNA with an expected size distribution of 400–9,000 bp on the TapeStation trace. Final sequencing libraries should show a characteristic peak around 500 bp with a size range of 400–700 bp (Figure 5). Sequencing at approximately 100,000 reads per nucleus on an Illumina NovaSeq S4 flow cell should provide sufficient depth for accurate cell-type annotation. After Cell Ranger processing and quality-control filtering, successful experiments should retain the majority of sequenced nuclei with low doublet rates and appropriate gene detection levels for downstream analysis. Supervised label transfer using SingleR should successfully annotate nuclei at the class, subclass, and subtype levels, with high-confidence assignments determined by delta scores.

## Limitations

This protocol has been validated in mouse primary visual cortex (V1) using five Cre-driver lines (Sepw1-Cre, Scnn1a-Cre, Tlx3-Cre, Npr3-Cre, and Ntsr1-Cre) targeting layer-specific excitatory neurons in young adult mice (P49-P70). Pilot testing is advised if applying START to other brain regions, developmental stages, or different Cre-driver lines. For brain regions without established reference transcriptomic atlases, generating a custom reference dataset from uninfected tissue will be necessary for accurate cell-type annotation.

Variability in AAV transduction efficiency and rabies glycoprotein expression between individual animals can result in large differences in the number of labeled input neurons per animal. This precludes quantitative comparisons of absolute input strength across different starter populations (i.e., different Cre lines). START analyses should therefore be limited to within-line comparisons of input cell-type proportions.

The 10-days rabies expression window allows sufficient time for retrograde viral spread to label monosynaptic input neurons and for mCherry expression to reach detectable levels for FACS sorting. This timepoint was chosen based on prior studies^2^ validating that rabies-infected neurons retain sufficient transcriptomic similarity to uninfected neurons for accurate cell-type assignment at 10 days post-infection. Shorter periods result in fewer labeled input neurons due to reduced retrograde spread and lower mCherry expression levels, while longer periods may result in greater rabies-induced transcriptional changes, though the extent to which this affects classification accuracy has not been systematically evaluated.

START depends on monosynaptic rabies tracing, which requires functional synaptic contacts for viral spread. Neurons that primarily use volume neurotransmission or have very sparse synaptic connections may be underrepresented in labeled input populations. Furthermore, it identifies transcriptomic cell types providing monosynaptic input but does not provide information about input neuron anatomical location, morphology, or synaptic strength. Tissue dissociation for nuclei isolation eliminates spatial information. For comprehensive circuit characterization, START should be combined with complementary approaches such as spatial transcriptomics, anatomical tracing, or electrophysiology.

A limitation of the current protocol is the reliance on fluorescence-activated nuclei sorting (FANS) to enrich rabies-labeled nuclei prior to sequencing. As with any sorting-based workflow, FANS introduces some degree of sample loss and physical stress on the sorted material, which may reduce the total number of recoverable nuclei. We adopted FANS for two principal reasons: first, it substantially reduces sequencing costs by excluding the large majority of unlabeled nuclei from the library. Second, and more critically, it provides the only reliable means of identifying rabies-infected nuclei when using snRNA-seq. Because rabies is a negative-sense RNA virus that replicates exclusively in the cytoplasm, its viral transcripts (including the mCherry reporter mRNA) are not reliably captured in the nuclear RNA fraction, making post-hoc computational identification of infected nuclei from snRNA-seq data unfeasible. Researchers who wish to bypass FANS and instead identify rabies-infected cells post hoc via viral transcript capture would need to adopt whole-cell scRNA-seq, as has been done in other tracing methods.^6^ However, this alternative introduces its own significant trade-offs including: (1) whole-cell scRNA-seq requires enzymatic dissociation of fresh tissue and is generally incompatible with frozen tissue, eliminating the flexibility to bank and batch-process samples; (2) enzymatic dissociation can introduce cell-type sampling bias^7^; and (3) prolonged enzymatic incubation at 37°C can induce immediate early gene and stress-response transcriptional artifacts that may confound downstream analyses.^7^ While emerging probe-based capture methods (e.g., 10× Chromium Flex) may eventually enable whole-cell profiling from fixed or frozen tissue, their application to rabies tracing has not been demonstrated. Users should evaluate these trade-offs based on their specific experimental goals and constraints.

Technical requirements include access to BSL-2 facilities for rabies work, stereotaxic injection equipment, FACS sorter, and 10× Genomics Chromium platform, which may limit accessibility for some laboratories.

## Troubleshooting

### Problem 1

Few or no mCherry-positive neurons visible in brain sections (related to Steps 46 and 51b).

### Potential solution

Successful rabies tracing should yield visible mCherry fluorescence in V1 sections under a dissection microscope. If no or very few mCherry-positive neurons are observed, this indicates a problem with viral injections or spread. Verify the helper AAV and rabies virus titers by performing test injections in control mice and examining expression after appropriate waiting periods. Confirm the correct Cre-driver mouse line genotype by PCR genotyping before beginning experiments. Validate stereotaxic coordinates by performing pilot injections with a fluorescent dye or tracer to confirm accurate V1 targeting. Ensure the full 3-weeks interval between AAV and rabies injections is maintained for adequate glycoprotein expression in starter cells. Check that the AAV and rabies virus was handled properly (stored at −80°C) and were not exposed to repeated freeze-thaw cycles, which reduce titer.

### Problem 2

Low yield of mCherry^+^ sorted nuclei (<1,000 nuclei) (related to Step 80).

### Potential solution

Typical experiments recover 1,000–5,000 mCherry^+^ nuclei per sample. Low recovery often results from nuclei adhering to the collection tube walls during FACS sorting. Ensure collection tubes are properly BSA-coated as described in the preparation steps (coat 0.2 mL PCR tubes with 5% BSA, vortex to coat walls, remove BSA, and air dry completely before adding collection buffer). Use small-volume PCR tubes (0.2 mL) rather than larger tubes (1.5 mL) as nuclei recovery is significantly better in smaller tubes with higher surface-to-volume ratios. Additionally, check the FACS gating strategy using uninfected control tissue to ensure mCherry-positive gate is appropriately set and not overly stringent. During micro-dissection, collect all visible mCherry-positive V1 tissue. If yields remain low despite proper tube coating, consider injecting larger volumes of virus (150 nL AAV and 300 nL rabies) in future experiments to increase labeling.

### Problem 3

Nuclei appear highly aggregated or clumped during FACS (related to Step 79).

### Potential solution

Nuclei aggregation causes frequent clogging during sorting and reduces sort purity. To prevent clumping, ensure nuclease-free BSA (50 μL of 10 mg/mL stock) was added to nuclei suspension and sample was kept on rotator or shaker at 4°C before sorting. Re-filter sample through pre-chilled 40 μm cell strainer immediately before loading onto FACS and re-filter every 20–30 min during extended sorts. If aggregation persists, reduce nuclei concentration by diluting the sample further with nuclei storage buffer.

### Problem 4

High mitochondrial percentage (>5%) or low gene detection (<500 genes per nucleus) in the sequencing data (related to Step 90).

### Potential solution

Poor RNA quality produces low-quality sequencing data unsuitable for cell-type annotation. Minimize the time from tissue thaw to 10× Chromium loading. Complete entire workflow within 3 hours. During nuclei isolation, work quickly on ice and ensure all buffers remain ice-cold. Verify the RNase inhibitor (RNasin Plus) was stored properly at −20°C and is not expired. Add protease inhibitors and DTT fresh immediately before use. Do not prepare buffers in advance. Reduce homogenization forces, use only the minimum strokes necessary (5 loose, 15 tight). Ensure tissue was flash-frozen immediately after dissection and stored continuously at −80°C without freeze-thaw cycles. During FACS, minimize nuclei time at 4°C. Sort as quickly as possible and load onto 10× Chromium immediately after sorting without freezing.

### Problem 5

Poor cell-type annotation confidence with low delta scores (related to Step 95).

### Potential solution

Low delta scores indicate uncertain cell-type assignments. First, verify that the reference dataset matches your experimental tissue. Use the mouse V1 atlas (GEO: GSE196771) for V1 experiments. Ensure the reference uses the same technology platform (10× v3 snRNA-seq). If sequencing depth was <100,000 reads per nucleus, re-sequence to 150,000–200,000 reads to improve gene detection. Validate that quality control was applied correctly: check that nuclei with <500 genes, >10,000 genes, or >5% mitochondrial transcripts were excluded. Confirm doublets were identified and removed using DoubletFinder. Try alternative supervised annotation methods (Seurat label transfer, scmap, CHETAH) to compare results. If using a brain region other than V1, ensure that you have an appropriate high-quality reference atlas for that region.

## Resource availability

### Lead contact

Further information and requests for resources and reagents should be directed to and will be fulfilled by the lead contact, Edward M. Callaway (callaway@salk.edu).

### Technical contact

Technical questions regarding execution of this protocol should be directed to and will be answered by the technical contact, Maribel Patiño (m3patino@health.ucsd.edu).

### Materials availability

This study did not generate new unique reagents.

### Data and code availability

Original snRNA-seq data generated in the associated study^1^ are available at Gene Expression Omnibus (GEO): GSE261436. Reference V1 snRNA-seq data used for annotation are available at GEO: GSE196771.

## Acknowledgments

We thank Michael Linglebach for suggesting the START acronym and staff members of the Salk Viral Vector Core, the Salk Flow Cytometry Core, and the Salk Next-Generation Sequencing Core. This work was supported by R34 NS116885 is National Institute of Neurological Disorders and Stroke (NINDS) and R01 EY022577 is National Eye Institute (NEI) (E.M.C.). M.P. was supported by NIH grant T32 GM007198, the Paul and Daisy Soros Fellowship for New Americans, 5R25MH101072-13, and the APA SAMHSA Fellowship. The Salk flow cytometry core was supported by the NIH-10.13039/100000054National Cancer Institute (NCI) Cancer Center Support Grant P30 014195 and Shared Instrumentation Grant S10-OD023689. C.C. was supported by a NIH/10.13039/100000025NIMH Blueprint D-SPAN Award (K00 MH132569), an NIH/10.13039/100000057NIGMS IRACDA Award (K12 GM068524), and the 10.13039/100000861Burroughs Wellcome Fund.

## Author contributions

M.P., S.K.B., and C.C. wrote the protocol, performed the experiments, and created the figures. E.M.C. supervised the project and edited the manuscript.

## Declaration of interests

The authors declare no competing interests.

## Declaration of generative AI and AI-assisted technologies in the writing process

During the preparation of this work the author(s) used Claude (Anthropic) in order to assist with grammar and language editing and to reformat table structures during document preparation. After using this tool/service, the author(s) reviewed and edited the content as needed and take(s) full responsibility for the content of the published article.
